# Perinatal oral exposure to low doses of bisphenol A, S or F impairs immune functions at intestinal and systemic levels in female offspring mice

**DOI:** 10.1186/s12940-020-00614-w

**Published:** 2020-08-31

**Authors:** Yann Malaisé, Corinne Lencina, Christel Cartier, Maïwenn Olier, Sandrine Ménard, Laurence Guzylack-Piriou

**Affiliations:** Toxalim (Research Centre in Food Toxicology), Université de Toulouse, INRA, ENVT, INP-Purpan, UPS, 31300 Toulouse, France

**Keywords:** Bisphenol A, Bisphenol S, Bisphenol F, Immune responses, Perinatal exposure, Intestine, Th1/Th17, Immunoglobulin, Cytokines

## Abstract

**Background:**

Bisphenol A (BPA), one of the highest-volume chemicals produced worldwide, has been identified as an endocrine disruptor. Many peer-reviewing studies have reported adverse effects of low dose BPA exposure, particularly during perinatal period (gestation and/or lactation). We previously demonstrated that perinatal oral exposure to BPA (via gavage of mothers during gestation and lactation) has long-term consequences on immune response and intestinal barrier functions. Due to its adverse effects on several developmental and physiological processes, BPA was removed from consumer products and replaced by chemical substitutes such as BPS or BPF, that are structurally similar and not well studied compare to BPA. Here, we aimed to compare perinatal oral exposure to these bisphenols (BPs) at two doses (5 and 50 μg/kg of body weight (BW)/day (d)) on immune response at intestinal and systemic levels in female offspring mice at adulthood (Post Natal Day PND70).

**Methods:**

Pregnant female mice were orally exposed to BPA, BPS or BPF at 5 or 50 μg/kg BW/d from 15th day of gravidity to weaning of pups at Post-Natal Day (PND) 21. Humoral and cellular immune responses of adult offspring (PND70) were analysed at intestinal and systemic levels.

**Results:**

In female offspring, perinatal oral BP exposure led to adverse effects on intestinal and systemic immune response that were dependant of the BP nature (A, S or F) and dose of exposure. Stronger impacts were observed with BPS at the dose of 5 μg/kg BW/d on inflammatory markers in feces associated with an increase of anti-*E. coli* IgG in plasma. BPA and BPF exposure induced prominent changes at low dose in offspring mice, in term of intestinal and systemic immune responses, provoking an intestinal and systemic Th1/Th17 inflammation.

**Conclusion:**

These findings provide, for the first time, results of long-time consequences of BPA, S and F perinatal exposure by oral route on immune response in offspring mice. This work warns that it is mandatory to consider immune markers and dose exposure in risk assessment associated to new BPA’s alternatives.

## Background

Bioaccumulation of Endocrine disruptors (EDs) in humans is suspected to cause adverse health effects [1]. Among EDs, bisphenol A (BPA) is widely used as a component of epoxy resins and polycarbonate plastics by industry. BPA is present in plastic food containers, metal cans as epoxy coatings, kitchenware toys, medical devices, and dental composites and sealants [2]. In humans, BPA has been shown to have developmental, reproductive, cardiovascular, immune, and metabolic adverse outcomes [3].

Indeed, based on the current estimations of infants total exposure to BPA via dietary and non-dietary sources, EFSA’s latest scientific opinion published in 2015 concluded that children and adolescents are over/above the temporary tolerable daily intake (TDI) of 4 μg/kg BW/d [4].

In 2017, BPA was identified as a very high concern substance in the list of the European Chemical Agency (ECHA). Regarding the recent regulations that further restrict the use of BPA in food contact materials [5], food packaging companies are exploring substitutes for the purpose of gradually eliminating BPA from their products [6].

BPA analogues such as BPS and BPF, which share the basic bisphenol structure, are increasingly used in the manufacturing of consumer products. BPS, which is more heat -and-photo-resistant than BPA, has been used in the production of polycarbonates and epoxy resins for the manufacturing of industrial and consumer products [7]. BPF is used in epoxy resins and coatings, especially for systems needing increased thickness and durability (i.e., high-solid/ high-build systems). BPF epoxy resins are used for several consumer products such as lacquers, varnishes, liners, adhesives, plastics, water pipes, dental sealants, and food packaging [8]. However, BPs are readily released from these products into the environment, contributing to human exposure through diet or drinking water [9]. Liao and Kannan (2013) observed the presence of BPA, BPF, and BPS (*N* = 267) in nine categories of foodstuffs, in the U.S.A and BPs were found in 75% of the food samples tested [10].

In humans, BPA, BPS and BPF cross the placenta and represent a risk for the foetus [11]. BPA exposure during perinatal period is associated with non-communicable diseases (NCDs) at childhood and adulthood [12–14]. Emerging evidence suggests that exposure to BPs, in particular BPA, is associated with an altered immune function.

In rodents, we showed that perinatal exposure to BPA increased the risk of food intolerance at adulthood [15], as well as the susceptibility to intestinal infection and/or to exacerbated mucosal inflammation by deregulating Th1/Th2 cytokine profiles [16].

Most of these in vivo studies addressed the developing immune system and only few studies have reported effects on the mature immune system. In adult mice, exposure to BPA did not affect oral tolerance but changed the Th1/Th2 polarization towards an increased Th1 immune response [17–20]. This effect of BPA on Th1/Th2 balance has also been reported in in vitro studies using murine splenocytes and T cells [21]. More recently, we showed that perinatal exposure to BPA induced intestinal and systemic immune imbalance in young adult offspring mice, through the modulation of splenic and intestinal Th1/Th17 immune responses [22, 23]. Our results also highlighted a sex-specific difference in the immune response of offspring after oral exposure of mothers to BPA. An increase in the development of Th17 cells in the offspring has also been described by Luo (2016) after gestational and lactational BPA exposure [24].

All these studies conclude that low doses of BPA interfere with the maturing immune system and provide information to consider for human health preservation [25]. However, few information is available concerning BPA’s analogues and their potential long-term consequences on the immune system. The considerable use of BPA analogues requires studies to better characterize their safety.

In this context, the objective of the present study was to compare the effects of oral exposure during the perinatal period (gestation and lactation) at two doses of BPA, BPS and BPF (5 and 50 μg/kg BW/d) on immune response at intestinal and systemic levels of adult female offspring mice. Indeed, humoral and cellular immunotoxic effects of BPA substitutes are poorly studied and are the object of this study.

## Methods

### Animals and BPA treatment

All experiments were approved by the Local Animal Care and Use Committee (TOXCOM 0035/EH-2013), in compliance with the European directive 2010/63/UE. To minimize desertion induced by handling during the perinatal period, C3H/HeN mice were used (Janvier, Roubaix, France). The study was conducted on more than three litters/treatment (supplementary data Fig. 1) and at least three animals born of each different litters were used for each measurement in order to minimize potential litter effect. The perinatal study was conducted as previously described [22]. Briefly, nulliparous female C3H/HeN mice were mated with male for 5 days and then individually isolated. Pregnant and lactating mice were treated orally daily from gestation day 15 to weaning of pups (post natal day 21; PND21) with 5 or 50 μg/kg BW/day of BPA, BPS, BPF or vehicle alone (0.1% ethanol in corn oil) as control group.

We chose to work with 5 μg/kg BW/day, a dose which is close to that 4 μg/kg BW/day estimated by EFSA in 2015 as tolerable daily intake (TDI) [4] and the dose of 50 μg/kg BW/day, corresponding to previous TDI (before 2015).

All groups were treated in parallel within the same timeframe and hosted in the same mouse house facility room. For clarity, groups were referred to as BPA5, BPA50, BPS5, BPS50, BPF5 and BPF50. All mice (mothers and offspring) were kept at a constant temperature (22+/− 1 °C) and maintained on a 12:12 h light/dark cycle (light on at 7:30 am). In the present study, we chose to work on female offspring based on previous studies showing that female immune responses are more sensitive to BPA exposure than male ones [26] and identifying which immunological endpoints are affected by BPA treatment [23]. Body weight (BW) was measured at PND10 and PND70. At PND70, female mice were euthanized by cervical dislocation, and blood, jejunum and feces were collected. *Lamina propria* from small intestine (si*LP*) and spleens were collected for primary cell culture.

### Humoral response in plasma and feces

Blood and feces were sampled. Intracardiac blood was collected with a heparinized syringe and recovered plasma was kept at − 80 °C. Fecal proteins were extracted mechanically in complete antiprotease cocktail (Roche Diagnostic, Meylan, France) and frozen at − 80 °C. Plasma and fecal IgG and IgA concentrations were measured by ELISA.

Plates were coated overnight at + 4 °C with 5 μg/ml sheep anti-mouse IgA (Sigma-Aldrich) or goat anti-mouse IgG (Southern Biotech, France) in PBS. Plates were blocked with PBS-5% fetal calf serum (FCS) (Invitrogen) before incubation with diluted samples or purified IgA or IgG (Southern Biotech). Horseradish-peroxidase (HRP)-conjugated goat anti-mouse IgA (Sigma-Aldrich) or goat anti-mouse IgG (Southern Biotech) were added, HRP was revealed using TMB (Becton Dickinson, France). Reaction was stopped adding H_2_SO_4_ 2 N and plates were analysed using automatic Infinite M200 microplate reader. Measurements were performed in duplicate and detection threshold correspond to at least three blank values.

### Immunoglobulin specificity against commensal *E. coli* lysate

Maxisorp 96-wells plates were coated with 5 μg/ml of protein from C3H/HeN isolated *E. coli* lysate, incubated with plasma (10 μg/mL IgG; 20 μg/mL IgA), and revealed as above-mentioned. Results were expressed as arbitrary units (AU) per 10 μg/mL of IgG ou 20 μg/ml of IgA, in comparison with a standardized immune serum. Measurements were performed in duplicate and detection threshold correspond to at least three blank values.

### Spleen and small intestine *lamina propria* (si*LP*) cells isolation

Spleens were collected and cells were isolated through 70 μm cell strainer to make a single-cell suspension in PBS-1% KnockOutTM SR (KO SR) (Gibco). Small intestines were washed in cold PBS, cut into 0.5 cm pieces, incubated four times in 30 ml of PBS 3 mM EDTA (Sigma-Aldrich) and digested in 20 ml of DMEM added with 20% FCS and 100 U/mL of collagenase (Sigma-Aldrich) for 40 min at 37 °C. Si*LP* cells were purified on a 40–80% Percoll gradient centrifuged for 15 min at 1800 x *g* at room temperature.

### Fluorescence-activated cell sorter analysis

Isolated cells from spleens and si*LP* were stained as follows: Regulatory T-cells: CD4 (BD), CD25 (BD), Foxp3 (ebioscience); Th17: CD3 (BD), RORγt (BD), IL-17 (BD) and Th1 (CD3 (BD), T-bet (BD) and IFN-γ (BD). The staining protocol was performed as previously described [23]. MACSQuant® Analyzers (Miltenyi Biotec) and VenturiOne® (AppliedCytometry) software were respectively used for data collection and analysis.

### Cytokines measurement

To culture, cells were seeded on 24-well plates at 1 × 10^6^ cells per well for cytokine assays in Cerrotini culture medium (Dulbecco modified Eagle medium supplemented with 8% Knockout serum replacement, (Gibco, Lifetechnologies, Paisley, UK), 36 mg/l asparagine, 116 mg/l arginine, 10 mg/l folic acid, 1 g/l 4-[2-hydroxyethyl]-1-piperazineethanesulfonic acid, 0.05 mmol/l β-mercapto-ethanol, 100 U/ml penicillin, 100 mg/ml streptomycin and 1 μg/ml fungizone)) in presence or absence of 5 μg/ml hamster anti-mouse CD3 and hamster anti-mouse CD28 (BD biosciences) coated wells. After 3 days of stimulation, culture supernatants were collected and frozen at − 80 °C prior to cytokines assay. Cytokines were measured in supernatant of primary cell culture of spleen or si*LP* by ELISA: IFN-γ and IL-17 present in primary cells culture supernatant were assayed using commercial enzyme linked immunosorbent assays (ELISA kits; Duoset R&D Systems, Lille, France). Cytokines were measured in feces suspended in RIPA buffer (0.5% deoxycholate, 0.1% SDS and 1% Igepal in TBS) containing complete anti protease cocktail (Roche). Fecal protein concentrations were measured using BCA uptima kit (Interchim). Lipocalin was assayed using commercial ELISA kits (R&D Systems). Measurements were performed in duplicate and detection threshold is defined for each molecule by the manufacturer’s instruction.

### Multivariate data processing

Mixomics package (6.8.2 version in RStudio software, Boston, MA, 1.0.44 version) was used to build first a principal component analysis based on the compilation of all data present in the study; secondly, a partial least-squares discriminant analysis (PLS-DA) was built to depict immune signature associated with BPs treatment. PLS-DA is a multivariate supervised approach that operates by projecting the samples (X) onto a low-dimensional space of so-called latent variables that maximizes the separation between different groups of samples according to their class labels (Y = mice BPs treatments). Repeated Mfold cross-validations were used to select the optimal number of latent variables for PLS-DA models with minimal error rate.

### Statistical analysis

Statistical analysis was performed using GraphPad Prism version 6.00 (GraphPad Software, San Diego, California, USA). Results were expressed as means +/− SEM. Kuskal-Wallis one-way ANOVA followed by Dunn’s post hoc test for multiple comparisons were used for statistical analysis. *P*-values < 0.05 were considered significant (indicated by asterisks in figures): *p < 0.05; **p < 0.01; ***p < 0.001; ****p < 0.0001.

## Results

### Body weight (BW) of offspring mice

**Fig. 1:**
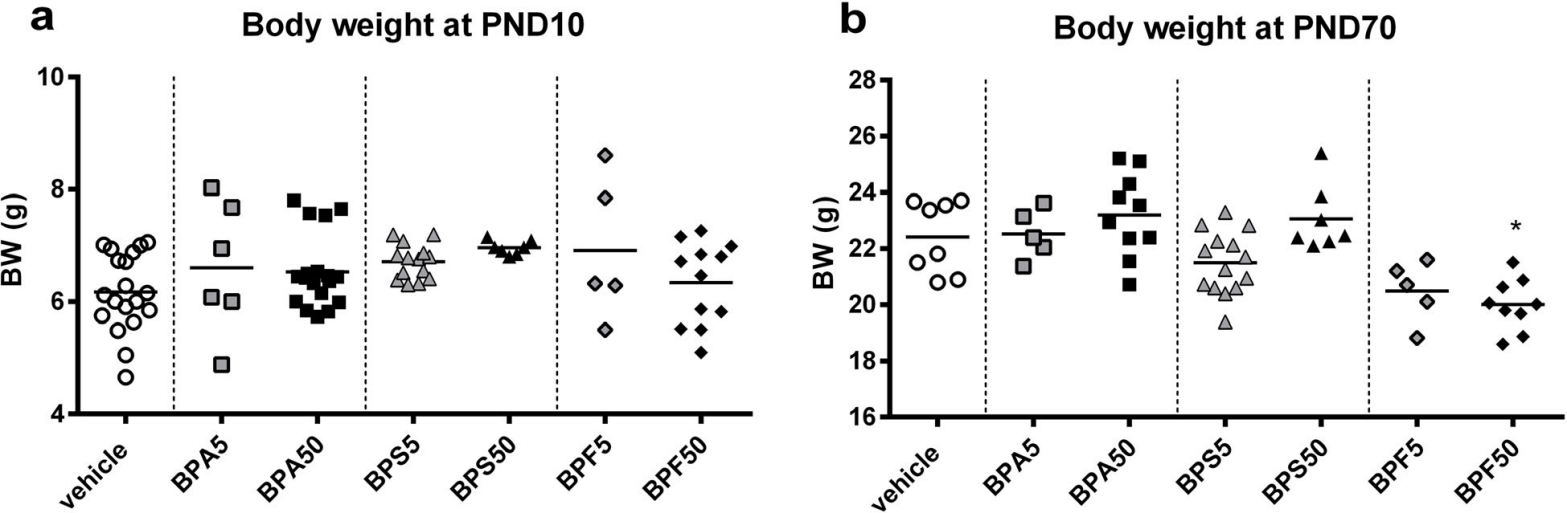
Body weight of young and adult offspring mice. **a** Body weight of female offspring mice in different treatment groups measured at PND10 and **b** at PND70. * *P* < 0.05 vs. vehicle group. *n* = 5–14

BPA, BPS and BPF after oral exposure did not provoke any change in BW of offspring female at PND10 (Fig. 1a). However a reduction in BW was observed in BPF group at PND70 (Fig. 1b), which is significant for BPF50 group (*p* < 0.05). The average number of offspring/litters for each treatment was dependent on the treatment with a lower number of progenies after BPA50 and BPS5 perinatal exposure (supplementary data Fig. 1a and b).

### Intestinal immune response of offspring mice

**Fig. 2:**
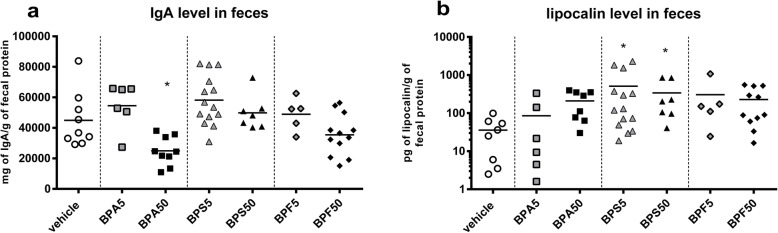
Perinatal exposure to bisphenols disrupts immune response in feces of adult offspring mice. **a** Total IgA concentration measured by ELISA in fecal samples of female offspring mice at PND70. **b** Lipocalin level determined in fecal supernatant of female offspring mice at PND70. * *P* < 0.05 vs. vehicle group. *n* = 5–14

**Fig. 3:**
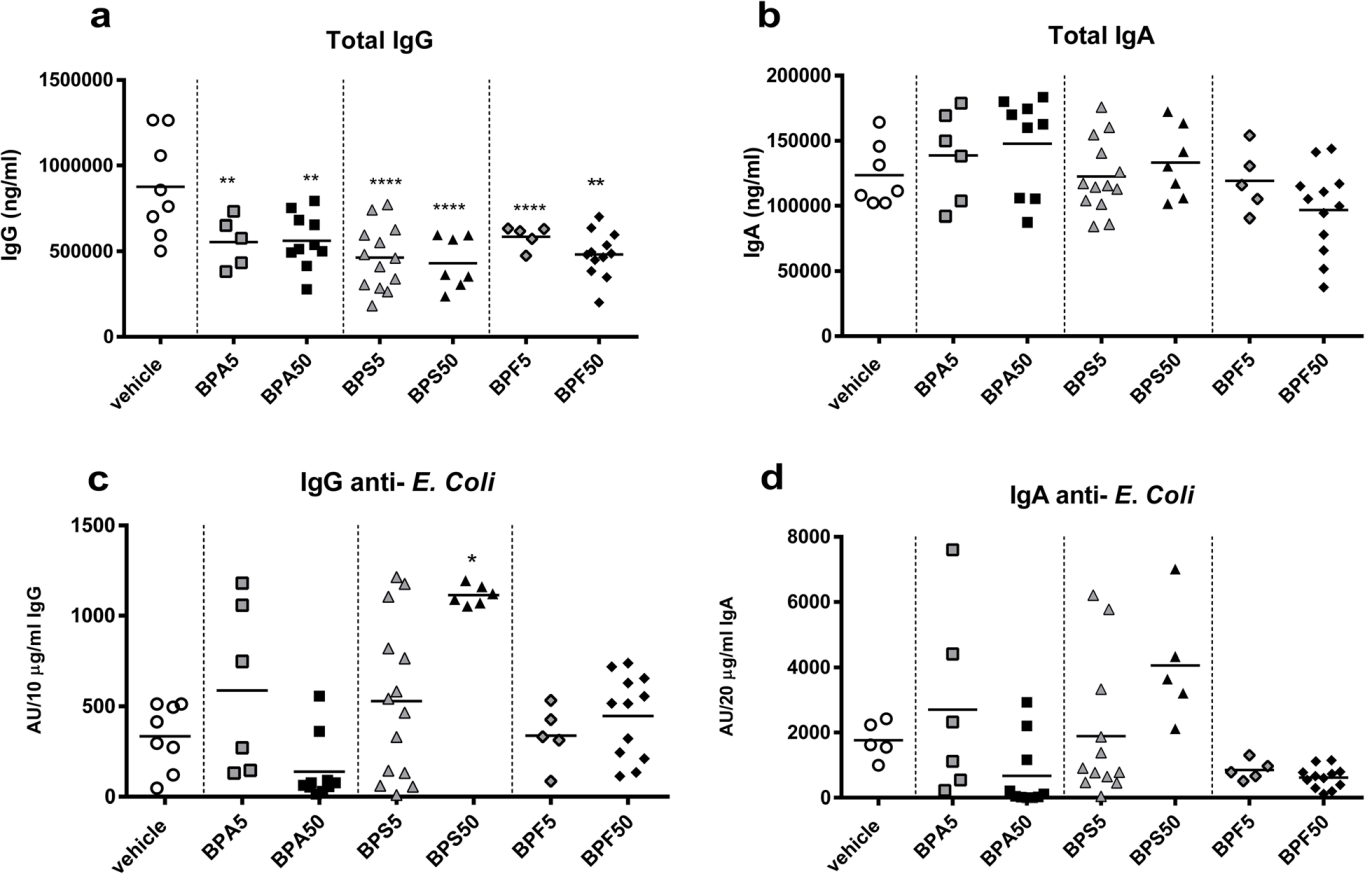
Perinatal exposure to bisphenols disrupts intestinal and systemic humoral responses in adult offspring dependent of bisphenol’s molecule. Plasma IgG (**a**) and IgA (**b**) concentrations measured by ELISA in female offspring mice at PND70. IgG (**c**) and IgA (**d**) specificity against *E. coli* lysate assessed in plasma by ELISA after normalizing to Ig concentrations. The lines represent the median (the 50th percentile). * *P* < 0.05; ** *P* < 0.01; **** *P* < 0.0001 vs. vehicle group. *n* = 5–14

In our previous work, we demonstrated a deleterious impact of mother oral exposure to BPA on intestinal barrier of offspring at PND50 [23]. Then, we wondered if oral exposure of mother with BPA, BPS or BPF has consequences on fecal IgA content in offspring female mice at adulthood. As previously demonstrated [23], we observed a significant reduction of fecal IgA level in BPA50 female offspring mice (*p* < 0.05) (Fig. 2a) associated with a slight but not significant increase of lipocalin level (Fig. 2b). Interestingly, lipocalin level in feces of BPS group, whatever the dose, was significantly increased (*p* < 0.05) (Fig. 2b). We noticed a significant decrease of plasmatic IgG (*p* < 0.01–0.005) in female offspring mice whatever the dose and BP used compared to vehicle group (Fig. 3a), without any change in total IgA levels (Fig. 3b). Only BPS50 offspring mice had a significant increase of specific anti-*E. coli* IgG in plasma compared to vehicle group (*p* < 0.05) (Fig. 3c). No difference in anti-*E. coli* IgA was observed (Fig. 3d).

### Intestinal and systemic cellular immune responses

**Fig. 4:**
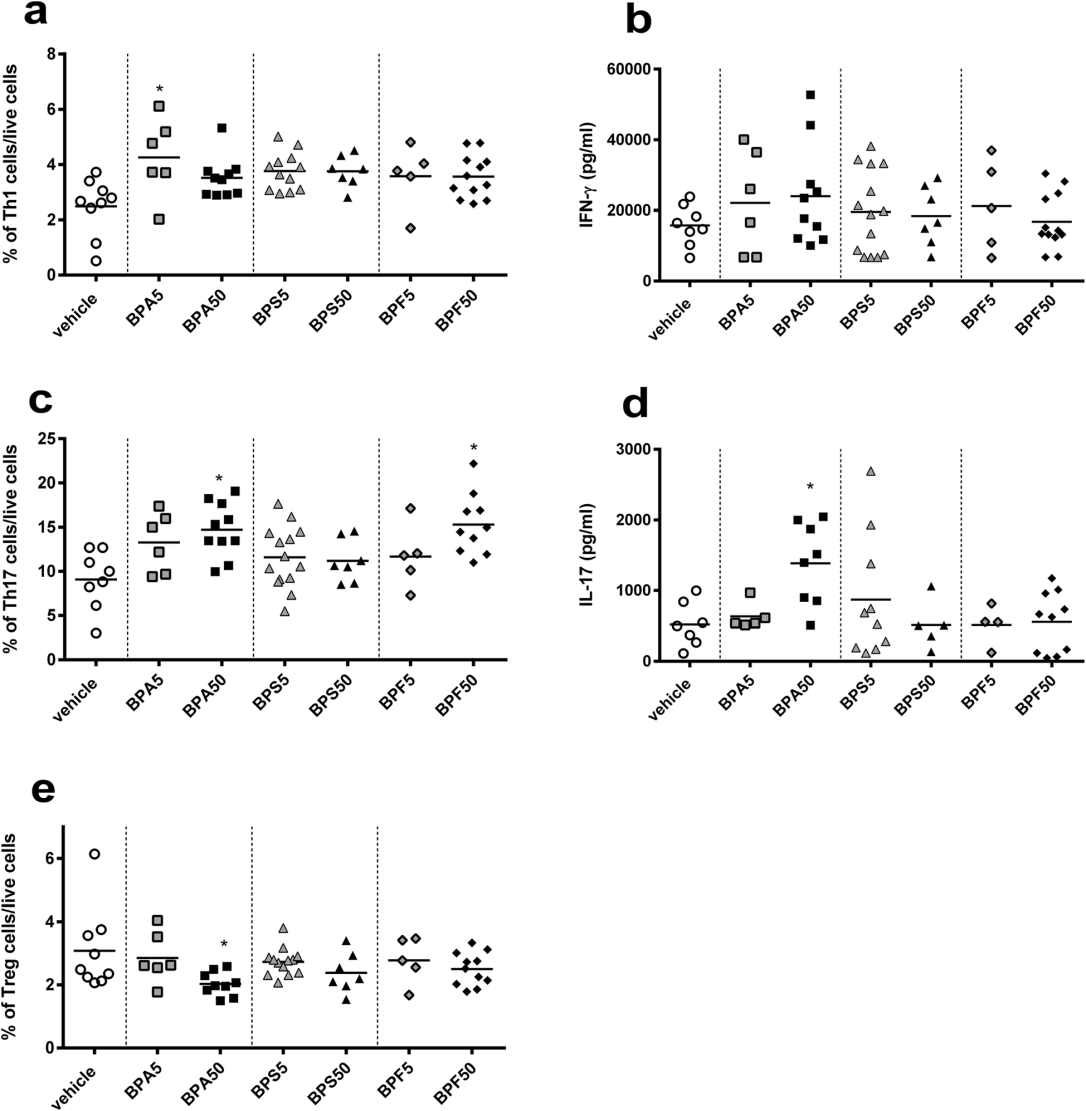
Perinatal exposure to BPA provokes intestinal Th1/Th17 immune response in adult offspring mice. Flow cytometry analysis of Th1 CD3^+^IFN-γ^+^T-bet^+^ (**a**) or Th17 CD3^+^RORγt^+^IL-17 ^+^ (**c**) lymphocytes from si*LP*. IFN-γ (**b**) or IL-17 (**d**) level assessed by ELISA after anti-CD3/CD28 in vitro restimulation of isolated lymphocytes from si*LP* offspring mice at PND70. (**e**) Proportion of CD4^+^CD25^+^FoxP3^+^ Treg cells in si*LP* of offspring mice at PND70. * *P* < 0.05 vs. vehicle group. *n* = 5–14

**Fig. 5:**
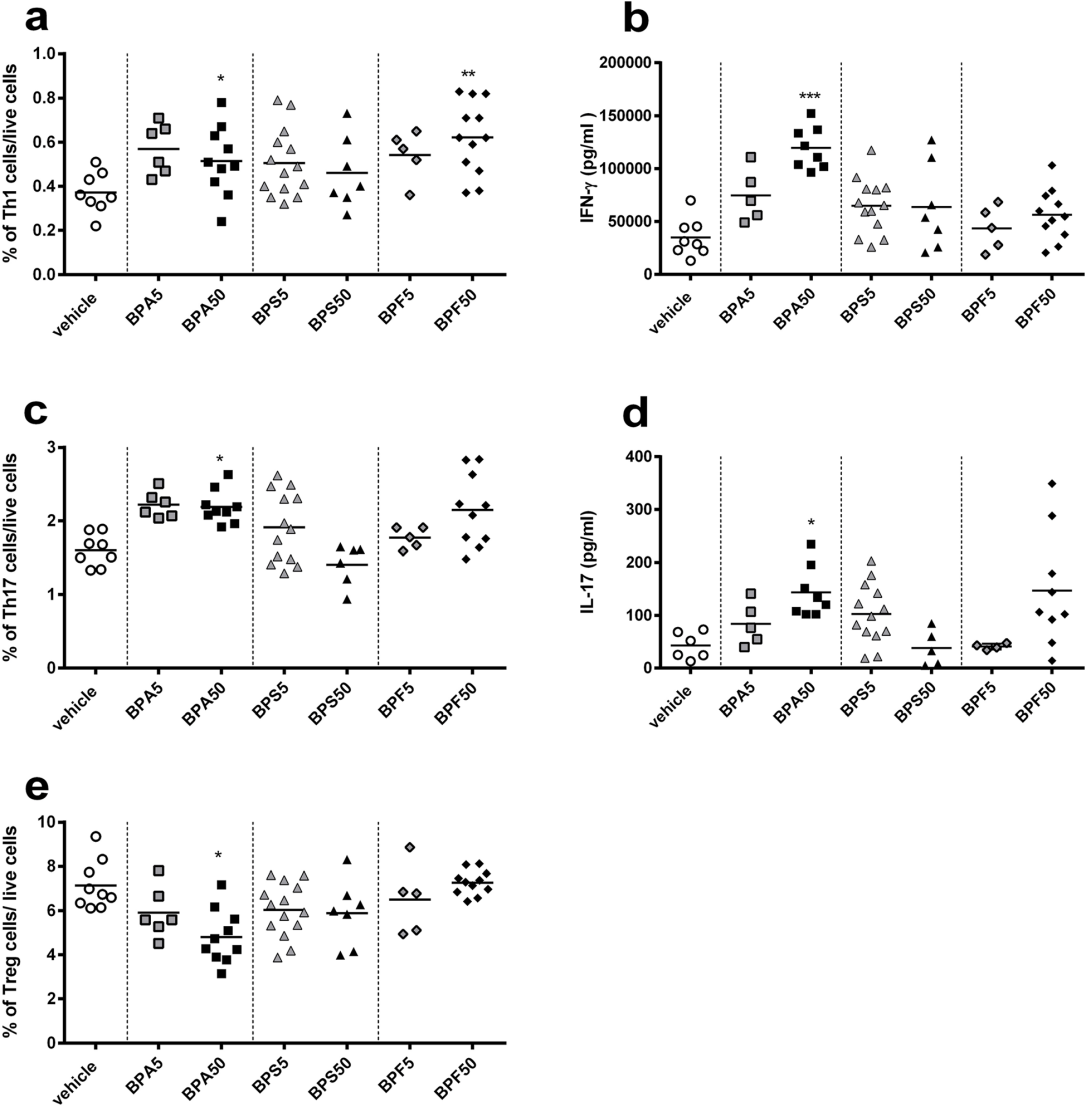
Perinatal exposure to BPA or BPF provokes systemic Th1/Th17 immune response in adult offspring mice. Flow cytometry analysis of Th1 CD3^+^IFN-γ^+^T-bet^+^ (**a**) or Th17 CD3^+^RORγt^+^IL-17^+^ (**c**) lymphocytes from spleen. IFN-γ (**b**) or IL-17 (**d**) level assessed by ELISA after anti-CD3/CD28 in vitro restimulation of isolated lymphocytes from splenic offspring mice at PND70. (**e**) Proportion of CD4^+^CD25^+^FoxP3^+^ Treg cells in spleen of offspring mice at PND70*.* * *P* < 0.05; ** *P* < 0.01; *** *P* < 0.001 vs. vehicle group. *n* = 5–14

First, we analyzed the frequency of T cells subsets at intestinal level. We observed an increase of Th1 (CD3^+^IFN-γ^+^T-bet^+^) subpopulation in *lamina propria* after perinatal oral exposure to BPA5 in female offspring mice, which is significant compared to vehicle group (*p* < 0.05) (Fig. 4a). This effect was associated with a slight but not significant increase of IFN-γ secretion in response to anti-CD3/28 in vitro restimulation (Fig. 4b). At intestinal level, a significant increase of Th17 (CD3^+^RORγt^+^IL-17^+^) frequency in BPA50 and BPF50 groups compared to vehicle group was noticed (*p* < 0.05), associated with an increase of IL-17 secretion in supernatant of si*LP* cells culture in response to TCR stimulation (anti-CD3/CD28) for BPA50 (*p* < 0.05) (Fig. 4c and d). Mother exposure to BPA50 via oral route during gestation and lactation induced a significant decrease (*p* < 0.05) in regulatory T cell (CD4^+^CD25^+^FoxP3^+^) frequency in si*LP* of offspring (Fig. 4e). All BP treatments after perinatal oral exposure provoked an increase of Th1 frequency in spleens of female offspring mice (Fig. 5a), which was significant for BPA50 and BPF50 (*p* < 0.05–0.01). Interestingly, we noticed a significant rise of IFN-γ secretion (*p* < 0.001) in response to anti-CD3/CD28 stimulation for BPA50 group (Fig. 5b). We also analyzed the Th17 frequency at systemic level. We observed a rise of Th17 frequency in BPA50 compared to vehicle group (Fig. 5c). Moreover, a significant increase of IL-17 secretion (*p* < 0.05) was noticed in splenocyte supernatants after anti-CD3/CD28 in vitro restimulation for BPA50 group compared to vehicle group (Fig. 5d). The level of IL-17 secretion was also increased for BPF50 offspring mice in comparison to vehicle group. Moreover, a significant decrease of regulatory T (Treg) cell frequency at systemic level (*p* < 0.05) in BPA50 female offspring mice was observed (Fig. 5e). The other BPs did not have any effect on Treg frequency.

### Discriminative parameters revealed through multivariate analyses

**Fig. 6:**
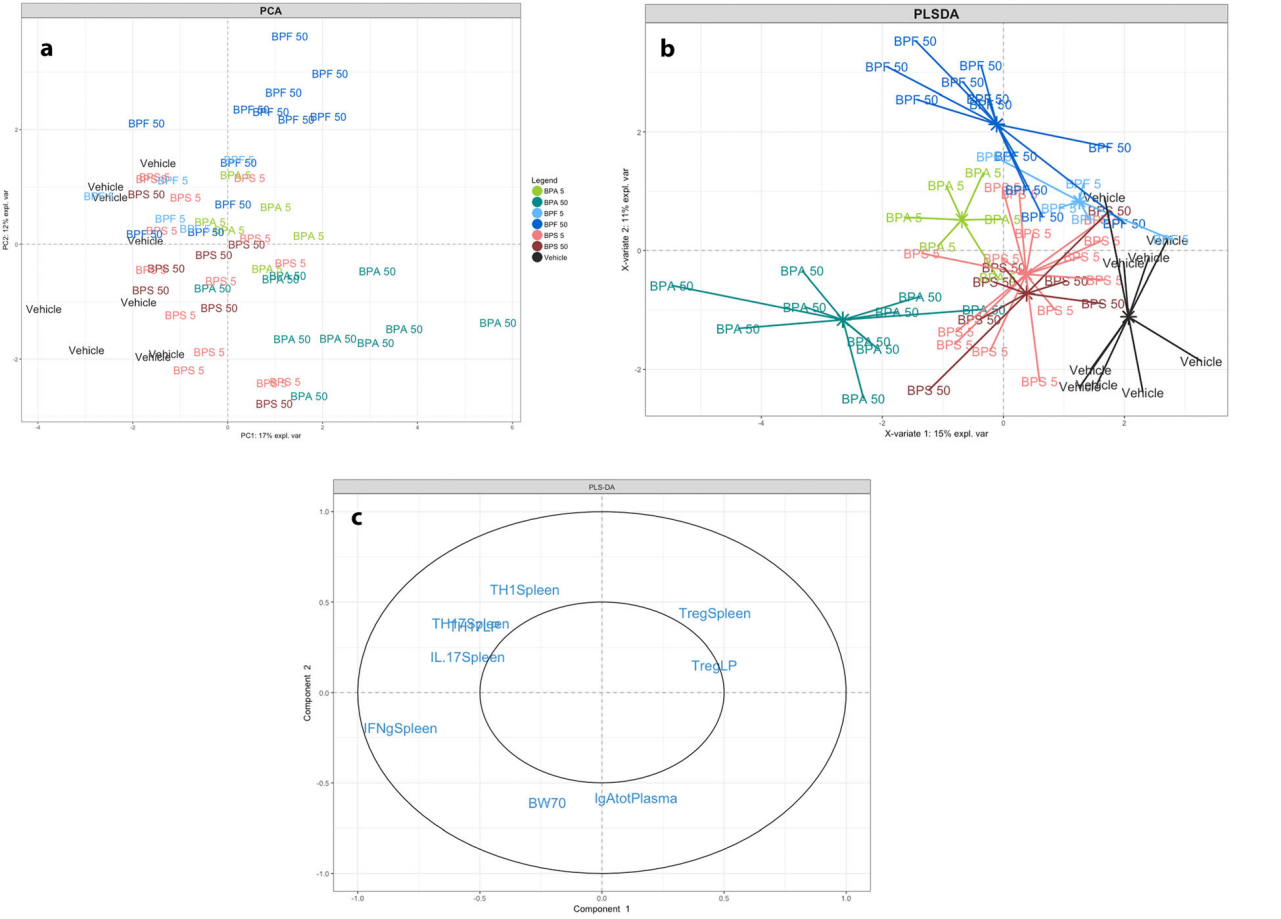
Multivariate analysis representing immune profiles in function of BP perinatal exposure in offspring mice. **a** Sample score plot and associated loading plot on the first two PCA components resulting from all data set in offspring mice. Each color indicated groups with 0.85% confidence level ellipse plots. **b** PLS-DA sample score plot and associated loading plot (**c**) on the first two components derived from data set from all treated groups in offspring mice. Only loadings with correlation threshold > 0.5 were represented on the loading plots. % expl var.: percentages for each first two components explained by the model. *n* = 5–14

Based on the compilation of all intestinal humoral and cellular immune responses associated data sets, a non-supervised method (Principal Component Analysis, PCA) was first performed to explore diversity patterns of responses according BP exposure and doses. Sample plot revealed a clear separation of mice according to the BP treatment and the dose used (Fig. 6a). The next step was used to perform a supervised analysis (PLS-DA) on female offspring data set in order to maximize observation of a host-response signature (related to immune response at intestinal and systemic level) characterizing each nature and dose of BP (Fig. 6b). The model used allowed us to discriminate the Vehicle group (black line) from treated BPs groups, BPA50 (dark green line), and BPF50 (dark blue line) treated mice being the most distant ones. BPA group (green line) even at low dose of 5 μg/kg BW/d showed a stronger separation from the vehicle group (Fig. 6b). The sample plot revealed a clear separation of BPA50 (dark green line), BPA5 (clear green line) and BPF50 (dark blue line) groups from the vehicle group (black line). The BPS50 (dark red line) group was less distant from the control group. The loading plot showed that IFN-γ level and IL-17 responses at systemic and intestinal levels and Treg cell frequency were important contributors to the separation between vehicle and BPA50 treated mice. Likewise, body weight and plasmatic IgA level contributed mainly to separate BPF treated mice from vehicle (Fig. 6c).

## Discussion

Since the ban of BPA use, the BPS and BPF are the most used BP as substitutes for BPA. A systematic review that included 32 studies (25 in vitro and 7 in vivo) revealed that BPF and BPS had similar hormonal effects than BPA [8]. The authors concluded that BPS and BPF seemed to have similar mechanisms of action than those of BPA, posing similar health issues. Other authors reported similarities in the toxicological profiles of BPS, BPF and BPA including metabolic, carcinogenic, and reproductive effects [27]. In the present study, we chose to work with 5 and 50 μg/kg BW/d by oral administration of mothers. Indeed, based on the current estimations of infants total exposure to BPA via dietary and non-dietary sources, EFSA’s latest scientific opinion published in 2015 concluded that children and adolescents are over/above the temporary tolerable daily intake (TDI) of 4 μg/kg BW/d [4]. The widespread contamination had led to high BP exposure in the general population through ingestion, inhalation and/or dermal absorption; however dietary exposure is considered as the primary pathway in humans [28]. In the present study, we analyzed the effects of two BPA substitutes, i.e. BPS and BPF in comparison to BPA after oral exposure of mothers. All the results of our study are summarized in Supplementary Figure 2. Environmental factor exposure during fetal or neonatal life can interact with the genome and the maturing immune system, and influence the onset of diseases in adulthood, including cancer, infertility, autoimmunity and metabolic disorders, a concept known as developmental origins of health and disease (DOHAD) [29]. We previously showed that gestational and lactational exposures to environmentally relevant doses of BPA caused adverse effects on the immune functions in offspring mice [16, 22], but no study investigated the effect of BPS and BPF and its consequences on the immune system of offspring mice. Here, we summarized and compared the impact of these different BPs on the intestinal and systemic immune functions (Supplementary Figure 2). During the neonatal period, the immune system, the intestinal epithelium and the microbiota form one entity, in which all parameters influence each other for their respective development until the equilibrium/homeostasis is reached. To achieve this stage, the immune system is first primed in utero by microbial metabolites of the mother, while high intestinal permeability at birth permits lumen-to-mucosa exchanges for further maturation of intestinal immune functions. The epithelial surface of the intestine plays a critical role in host protection. Intestinal IgA is involved in the development and maintenance of the homeostasis between microbiota and the host immune system [30]. Interestingly, our results demonstrated that perinatal exposure to BPA50 induced a fall of fecal IgA in offspring adult mice, but no effect was observed after BPS and BPF exposure. These results are in accordance with our previous studies showing a reduced IgA production after perinatal exposure of BPA [23]. However, a significant increase of lipocalin level, an inflammatory marker, in feces was detected after BPS perinatal exposure at both doses 5 and 50 μg/kg BW/d, highlighting the fact that the effect of this bisphenol on intestinal immune response involves a different mechanism compared to BPA. At plasmatic level, we observed an increase of anti-*E. coli* IgG in offspring after mother’s exposure to BPA5 and BPS50. We obtained similar results after oral administration of BPA in female offspring mice [23]. Interestingly, the low dose of BPS provoked an increase of anti-*E. coli* IgG in offspring mice correlated with high lipocalin level in feces, adding evidence of im-paired intestinal immune barrier in offspring mice ex-posed to BPA analogues.

In the gut, CD4^+^ T cells contribute to immunity by differentiating into various subsets, notably inflammatory (Th17/Th1) and regulatory T cells (Treg), Th17 cells being the most abundant CD4^+^ T cells in mucosal tissues. They secrete isoforms of IL-17 and/or IL-22, which confer protection against fungi and pathogenic bacteria. Our study reveals the ability of BPA and BPF to provoke a sharp increase in Th1 and Th17 frequency associated with an increase of IL17 and IFN-γ level production after in vitro anti-CD3/CD28 restimulation of intestinal immune cells of female offspring mice. These results are in agreement with our previous studies demonstrating that perinatal exposure to BPA after oral administration induced a potent Th1/Th17 signature at local level [23]. Interestingly, in the present study, Th1/Th17 cytokines production are stimulated even by low dose of BP i.e. 5 μg/kg BW/d. At the systemic level, we also reported an increase of IFN-γ and IL-17 cytokines production after anti-CD3/CD28 restimulation of splenocytes from BPA and BPF-exposed offspring mice. This effect was associated with an increment of Th1 and Th17 frequency but only with higher BP dose (50 μg/kg BW/d). Luo et al. (2016) recently reported similar observations after gestational and lactational exposures to BPA [24]. Others studies revealed an imbalance in immune responses after exposure of pregnant female rodents to varying relevant human-exposure levels of BPA [15]. These studies reported an increase in a pro-inflammatory Th1 response in the offspring. It is well known that immune tolerance requires the participation of Treg cells [31]. We observed a decrease in Treg cells isolated from si*LP* or spleen only in offspring mice exposed to BPA50. This result is in accordance with those obtained by Malaisé et al. (2018) in female offspring mice after BPA perinatal exposure by oral route [23].

This study compared for the first time, the effect of three bisphenols on the immune response at intestinal and systemic levels in adult female offspring after oral perinatal exposure. It reveals a specific effect of BPS on IgG response toward commensal microbiota (anti- *E. coli*). Gestational and lactational exposure to BPA and BPF were found to induce prominent changes in female offspring mice in intestinal and systemic cellular immune responses, inducing an intestinal Th1/Th17 inflammation. Our findings reveal that perinatal exposure to environmentally relevant doses of BPA and BPF results in changes of Th1 and Th17 development, which may contribute to developmental immunotoxicity. In fact, IL-17-secreting Th17 cells are key players to promote inflammatory diseases in mice [32]. Strong evidence revealed that Th17 cells represent a distinct subset of CD4^+^ T lymphocytes that plays a critical role in chronic inflammation and autoimmunity in mice [33]. Indeed, while the pro-inflammatory properties of IL-17 are key to its host-protective capacity, unrestrained IL-17 signaling is associated with immunopathology, autoimmune diseases and cancer progression [34].

## Conclusions

These experimental findings warrant further epidemiological studies to assess the effects of BPA and BPF burden in mothers on the risk of developing childhood and adult immune-mediated diseases in the female offspring mice. An uncontrolled acceleration of the system or failure of the brakes can both lead to persistent inflammation resulting in tissue damage and NCDs later on. We demonstrated that BPA substitutes BPS and BPF after gestational and lactational exposures are able to affect intestinal and systemic immune responses of adult offspring mice, at both 5 and 50 μg/kg BW/d involving different or similar mechanisms compared to BPA, questioning their safety and the rationale of their use to replace BPA. To conclude, not only BPA but also its substitutes BPS and BPF have immunotoxic effects on offspring mice at intestinal and systemic levels.

## Supplementary information

**Additional file 1 **: **Figure S1.** Consequence of oral exposure to bisphenols on birth rate of offspring mice. **(a)** Mean of number of offspring and sex in vehicle, BPA, BPS and BPF groups (male: grey box; female: black box). Numbers at top of each column correspond to the number of mothers used for each treatment group. (**b)** Offspring female number for each group used in the study. *n* = 5–14.

**Additional file 2 **: **Figure S2.** Summary tables of the study cohort characteristics.

## Data Availability

Please contact the corresponding authors with all requests.

## Abbreviations

BP: Bisphenol
BW: Body weight
d: Day
E: Escherichia
ED: Endocrine disruptor
ELISA: Enzyme linked immunosorbent assay
FCS: Fetal calf serum
Ig: Immunoglobulin
IL: Interleukine
PLS-DA: Partial Least-Squares Discriminant Analysis
PCA: Principal Component Analysis
PND: Postnatal day
siLP: Small intestine lamina propria
Th: T helper; Treg regulatory T
