# Supplementary figures and images for: Perinatal oral exposure to low doses of bisphenol A, S or F impairs immune functions at intestinal and systemic levels in female offspring mice
Source: Environ Health. 2020 Aug 31;19:93. doi: 10.1186/s12940-020-00614-w (PMC7457519; doi:10.1186/s12940-020-00614-w)

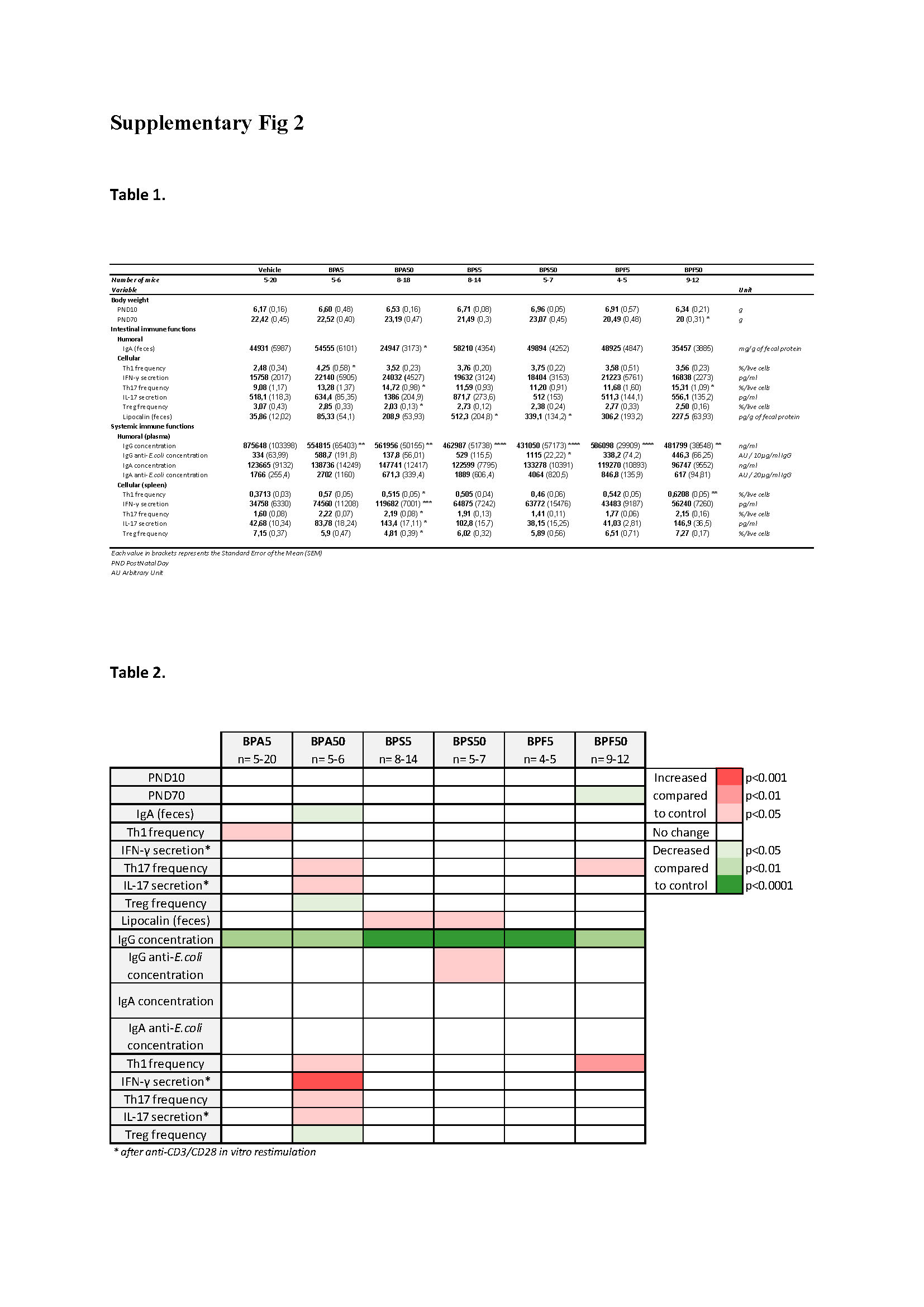

Supplement: Supplementary file 2 — Additional file 2 : Figure S2. Summary tables of the study cohort characteristics. [file 12940_2020_614_MOESM2_ESM.jpg]
